# Reflections on causes of suicidal behaviour

**DOI:** 10.1017/S2045796018000562

**Published:** 2018-10-16

**Authors:** U. Hegerl, I. Heinz

**Affiliations:** 1 Department of Psychiatry and Psychotherapy, Medical Faculty, University Leipzig, Semmelweisstr. 10, D-04103 Leipzig, Germany; 2 European Alliance against Depression, Semmelweisstr. 10, D-04103 Leipzig, Germany; 3 German Alliance against Depression, Semmelweisstr. 10, D-04103 Leipzig, Germany

**Keywords:** Depression, models/theories of psychiatry, stressful life events, suicide

According to the suicide report published by the World Health Organization (WHO, 2014), more than 800 000 people died by suicide in 2012 and the number of attempted suicides is estimated to be about 20 times higher. In Europe and most other countries, suicide rates are higher for males than females and increase with age. Attempted suicide rates are higher for females. The higher lethality of suicidal acts for males results from the choice of more lethal suicide methods (e.g. hanging, use of fire arms) whereas intoxication is by far the most preferred method for females (Cibis *et al*., 2012). However, even within the same method, the lethality is higher for males indicating that also other factors such as intentionality, reduced help-seeking or the influence of alcohol or drugs contribute to theses gender differences (Mergl *et al*., 2015).

Every suicide is the result of deep suffering and leaves behind traumatised relatives. In most cases, suicides are not an act of free will but occur in the context of depression and other psychiatric disorders. To prevent suicidal behaviour is an important task for all societies.

When discussing the best strategy for achieving this aim, it is important to have a clear understanding of the causes of suicidal behaviour. Two explanatory models describing the causal relationships between psychosocial factors, depression and suicides are displayed in Fig. 1.

**Fig. 1.**
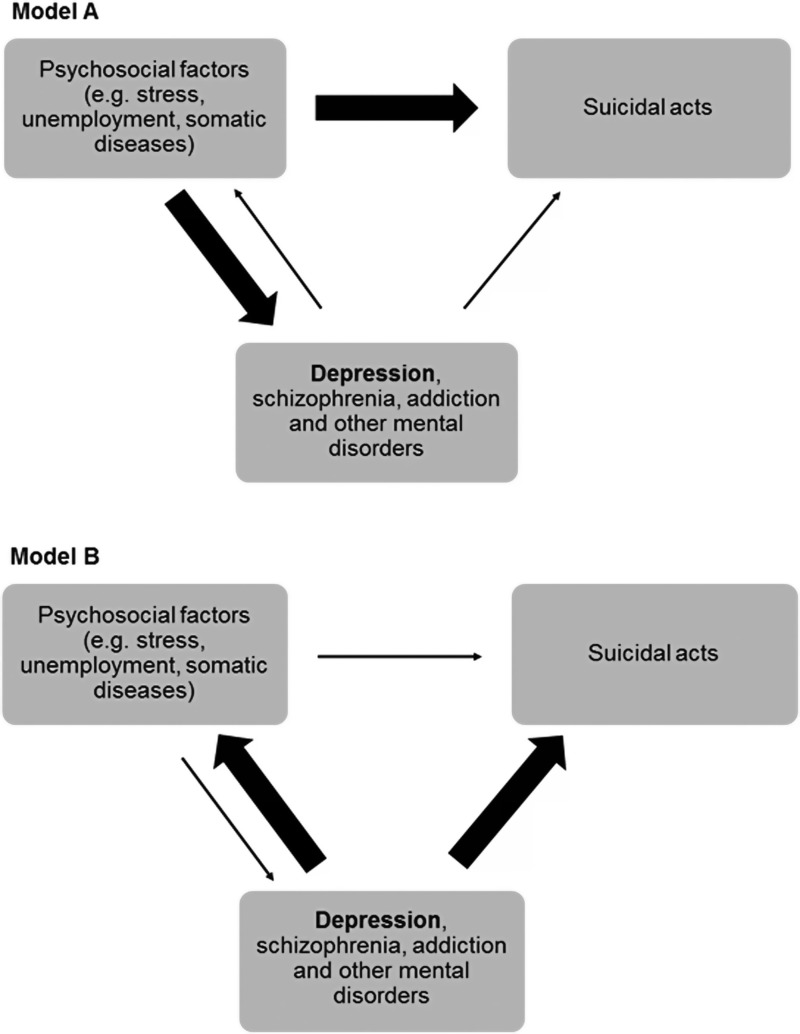
Models of causal relationships between psychosocial factors, depression and other mental disorders, and suicidal behaviour (Hegerl *et al*., 2015).

Model A assumes that social determinants (e.g. social cohesion, unemployment rate), individual factors such as stressful life circumstances (e.g. unemployment, interpersonal conflicts, health problems) or other life adversities are major causes for both psychiatric disorders and suicidal behaviour. Model B attributes a stronger causal role to depression and other psychiatric disorders which are seen as major causes for suicidal behaviour as well as for a variety of life adversities (e.g. unemployment, divorce, somatic disorders). Model A is favoured by lay people, sociologists, but also many professionals in the health care system and by health politicians. Experienced psychiatrists, however, often are inclined to model B. Psychiatrists are in the special position to have daily and close contact with depressed and acutely suicidal patients and, in countries where all patients after an attempted suicide are seen by an psychiatrist, they have a representative view on the phenomenon of suicidal behaviour. Both models clearly differ concerning what appears to be the most promising strategy for suicide prevention: In model A, public health interventions will appear promising; in model B, improving the care of people with depression and other mental disorders should be a central element in suicide prevention. In the following, some thoughts and arguments will be presented on why in the view of the authors, model B is the more valid one:

(1) There are several factors which contribute to the tendency to overestimate external and psychosocial factors as causes for depression and suicidal behaviour (as assumed in model A):
  • Lay people understandably assume that depression is more or less the same as what they have felt as a reaction to life adversities, e.g. the end of a relationship or severe somatic disorders. However, moderate-to-severe depression differs subjectively and objectively from a reaction to difficult life circumstances. Patients consistently report that what they felt within a depressive episode was qualitatively different. Also for experienced psychiatrists, several qualitative aspects of the symptomatology allow to differentiate an understandable reaction to the bitterness of life from a depressive episode. For example, the tendency to blame oneself (feelings of guilt), emotional numbness (‘feeling of nothing’), high inner tension (‘feeling as being permanently before an examination’), symptoms of delusion (e.g. topics of poverty or guilt), responding to sleep deprivation, suicidal impulses and also a positive family history regarding depression or earlier depressive episodes are pointing to a depressive disorder.
  • If a depressive episode starts, the following regularly happens: depression ‘is looking around’ for negative aspects and in everybody's life always something is found. This negative aspect is then enlarged, tied to hopelessness and put in the centre of being. If someone is, e.g. suffering from lower back pain or tinnitus, these somatic complaints will be perceived as unbearable and stated as causes for suicidal thoughts. However, when depression is treated, these complaints are still present but are now well tolerated. The same happens for all negative aspects in life such as stress at work, interpersonal conflicts or financial problems. If depression is treated, hope and energy return and problems are downsized to part of normal life.
  • A tendency to perceive feelings of guilt is a typical symptom of depression. Therefore, the patients tend to blame themselves for their depressed state and do not perceive themselves as a victim of a medical disorder needing professional help.
(2) Suicides are rarely committed by free will, but are the negative consequences of, in most cases, untreated psychiatric disorders. Psychological autopsy studies in western countries showed that around 90% of suicide victims had been suffering from psychiatric disorders (Cavanagh *et al*., 2003; Arsenault-Lapierre *et al*., 2004). Most important in this context are affective disorders, which have been present in more than 50% of suicide victims (Cavanagh *et al*., 2003; Lönnqvist, 2009). Furthermore, schizophrenia, substance-related and personality disorders were associated with increased suicide risk (Bertolote *et al*., 2004).
(3) An association between economic crisis in different countries and increases in suicide rates has been observed (Chang *et al*., 2013; Nordt *et al*., 2015) and societal stress including unemployment and financial problems were seen as a plausible explanation for this relationship. However, this might not be the correct interpretation. During the economic crisis starting in 2008, mental health services have been shut down in many countries, and in those without a general health insurance system where patients have to pay for e.g. antidepressants or psychotherapy, it can be assumed that more suicidal patients with depression and other psychiatric disorders remained untreated. This interpretation is supported by a recent publication of the European Alliance against Depression (http://www.eaad.net; Gusmão *et al*., 2013). Data from 29 European countries have shown that changes in suicide rates were not related to economy (Global Domestic Product), unemployment rates or alcohol consumption, but to the prescription of antidepressants and to a lesser degree to divorce rates. An increase in prescription of antidepressants was associated with a decrease in suicide rates. This does, however, not allow the conclusion that antidepressants have directly developed antisuicidal effects. The prescription rate for antidepressants could be a proxy marker for seeking professional help and for, e.g. getting out of isolation.
(4) After the reunification of Germany in 1990, huge social stress was induced in the Eastern part of Germany. Unemployment rate increased from 0 to 20%, societal values were changed and professional careers broken. According to the sociologist Durkheim (Durkheim, 1983), this would be a classic scenario leading to increases in suicide rates. However, the opposite, namely a sharp decrease in suicide rates was found in the Eastern part of Germany. Detoxification of house gas and a reduction in autopsy rates occurring after the reunification in the Eastern part of Germany might have contributed to some degree to this decline in suicide rates, but are not sufficient factors to explain it. This decline in suicide rates in the Eastern part of Germany after the reunification can be considered as a quite convincing falsification of Durkheim's theory.
(5) If a patient diagnosed with cancer or another severe somatic disorder is despaired and commits suicide, many people will tend to assume that these illnesses have caused the suicide. However, we should be cautious with such conclusions, even if the patient has stated them as the reason for his suicidal tendencies. In a large study in the UK, patient records of more than 4 million primary care patients were collected (Webb *et al*., 2012). Over several years, 873 suicides were documented. Hence, it was possible to analyse how many of the suicide victims were affected by at least one severe somatic disorders (stroke, cancer, asthma, cardiovascular disease, diabetes mellitus, chronic obstructive pulmonary disease, epilepsy, chronic lower back pain, osteoporosis, osteoarthritis). It was found that 38.7% of suicide victims had at least one of these somatic disorders. However, this was also the case in 37% of the 17 000 controls who did not commit suicides. When looking at the diagnosis of cancer: 3.4% of the suicide victims were diagnosed; however, the same applied to 3.2% of the control group. According to these data, severe somatic disorders do not strongly increase the suicide risk. It is hard to see how any bias can have flawed these results.

These are examples for arguments supporting model B. Improving the care of depression and other psychiatric disorders becomes then a central element in suicide preventive strategies. In line with this reasoning, the four-level community-based intervention concept of the European Alliance against Depression has been developed, which combines the aims to prevent suicidal behaviour and to improve care for people with depression. It has proven to be effective in preventing suicidal behaviour in different European regions (Hegerl *et al*., 2013).

Models A and B are not mutually exclusive, but they reflect differences in the emphasis given to different causal factors. A shift to model B will have consequences going beyond the design of suicide prevention interventions:

• If depression is not seen as secondary to difficult life circumstances, but as an independent disorder as any other disorder, depression and also suicidal behaviour will gain relevance as target for resource allocation in the health care system and other areas. Model B will help to see the many completed and attempted suicides not as a partly understandable reaction to the ‘bitterness of life’, but as what they are: the tragic outcome of an, in most cases, not optimally treated psychiatric disorder. This will help to understand that the same efforts have to be made to prevent suicidal behaviour as to prevent, e.g. traffic accidents.
• In German studies (Liwowsky *et al*., 2009; Bühler *et al*., 2013), it was found that about 40% of long-term unemployed persons suffer from affective disorders, but <10% receive treatment according to national treatment guidelines (Deutsche Gesellschaft für Psychiatrie, Psychotherapie und Nervenheilkunde and Ärztliches Zentrum für Qualität in der Medizin, 2015). However, having model A in mind, the staff in job centres does not usually perceive these disorders as removable obstacles on the way back to work, but more as a consequence of the stress and the frustration associated with being unemployed. In fact, in line with model B, in many cases, depression and other mental disorders are the reasons for getting unemployed and for having difficulties getting back to work. The concept of ‘Psychosocial Coaching’ developed in model projects in job centres in Munich and Leipzig addressed this situation. Trained psychologists provide structured diagnostics to unemployed with mental problems and, if necessary, guide them to a regular treatment in the health care system.
• It can be argued that model B, which sees depression as an independent disorder involving disturbed brain functions associated with suicidal behaviour, leads to increased stigmatisation. However, clinical experience often shows the contrary: patients feel relieved as soon as they understand that they are suffering from a disorder which can hit everybody. This helps them to not blame themselves for being unable to deal with stressful life circumstances.

## References

[ref1] Arsenault-Lapierre G, Kim C and Turecki G (2004) Psychiatric diagnoses in 3275 suicides: a meta-analysis. BMC Psychiatry 4, 37. 15527502 10.1186/1471-244X-4-37 PMC534107

[ref2] Bertolote JM, Fleischmann A, de Leo D and Wasserman D (2004) Psychiatric diagnoses and suicide. Revisiting the evidence. Crisis 25, 147–155. 15580849 10.1027/0227-5910.25.4.147

[ref3] Bühler B, Kocalevent R, Berger R, Mahler A, Preiß B, Liwowsky I, Carl P and Hegerl U (2013) Versorgungssituation von Langzeitarbeitslosen mit psychischen Störungen. Der Nervenarzt 84, 603–607. 23052889 10.1007/s00115-011-3457-6

[ref4] Cavanagh JTO, Carson AJ, Sharpe M and Lawrie SM (2003) Psychological autopsy studies of suicide: a systematic review. Psychological Medicine 33, 395–405. 12701661 10.1017/s0033291702006943

[ref5] Chang SS, Stuckler D, Yip P and Gunnell D (2013) Impact of 2008 global economic crisis on suicide. Time trend study in 54 countries. British Medical Journal 347, 5239. 10.1136/bmj.f5239 PMC3776046 24046155

[ref6] Cibis A, Mergl R, Bramesfeld A, Althaus D, Niklewski G, Schmidtke A and Hegerl U (2012) Preference of lethal methods is not the only cause for higher suicide rates in males. Journal of Affective Disorders 136, 9–16. 21937122 10.1016/j.jad.2011.08.032

[ref7] Durkheim É (1983) Der Selbstmord, Suhrkamp-Taschenbuch Wissenschaft. Suhrkamp: Frankfurt am Main.

[ref8] Gusmão R, Quintão S, McDaid D, Arensman E, van Audenhove C, Coffey C, Värnik A, Värnik P, Coyne J and Hegerl U (2013) Antidepressant utilization and suicide in Europe. An ecological multi-national study. PLoS ONE 8, 66455. 10.1371/journal.pone.0066455 PMC3686718 23840475

[ref9] Hegerl U, Rummel-Kluge C, Värnik A, Arensman E and Koburger N (2013) Alliances against depression – a community based approach to target depression and to prevent suicidal behaviour. Neuroscience and Biobehavioral Reviews 37, 2404–2409. 23438891 10.1016/j.neubiorev.2013.02.009

[ref10] Hegerl U, Koburger N and Hug J (2015) Depression und Suizidalität. Nervenheilkunde 34, 900–905.

[ref11] Liwowsky I, Kramer D, Mergl R, Bramesfeld A, Allgaier AK, Pöppel E and Hegerl U (2009) Screening for depression in the older long-term unemployed. Social Psychiatry and Psychiatric Epidemiology 44, 622–627. 19048174 10.1007/s00127-008-0478-y

[ref12] Lönnqvist J (2009) Major psychiatric disorders in suicide and suicide attempters In Wasserman D and Wasserman C (ed.) Oxford Textbook of Suicidology and Suicide Prevention. Oxford: Oxford University Press, pp. 276–286.

[ref13] Mergl R, Koburger N, Heinrichs K, Székely A, Tóth MD, Coyne J, Quintão S, Arensman E, Coffey C, Maxwell M, Värnik A, van Audenhove C, McDaid D, Sarchiapone M, Schmidtke A, Genz A, Gusmão R and Hegerl U (2015) What are reasons for the large gender differences in the lethality of suicidal acts? An epidemiological analysis in four European countries. PLoS ONE 10, 0129062. 10.1371/journal.pone.0129062 PMC4492725 26147965

[ref14] Nordt C, Warnke I, Seifritz E and Kawohl W (2015) Modelling suicide and unemployment. A longitudinal analysis covering 63 countries, 2000–11. The Lancet. Psychiatry 2, 239–245. 26359902 10.1016/S2215-0366(14)00118-7

[ref15] S3-Leitlinie/Nationale. VersorgungsLeitlinie Unipolare Depression – Langfassung, 2. Auflage. DGPPN, BÄK, KBV, AWMF. Available at http://www.depression.versorgungsleitlinien.de (Accessed 24 July 2018).

[ref16] Webb RT, Kontopantelis E, Doran T, Qin P, Creed F and Kapur N (2012) Suicide risk in primary care patients with major physical diseases. A case-control study. Archives of General Psychiatry 69, 256–264. 22393218 10.1001/archgenpsychiatry.2011.1561

[ref17] World Health Organization (2014) Preventing Suicide: A Global Imperative. Geneva, Switzerland: World Health Organization.

